# Quercetin Zinc and Iron Metal Complexes Protect against Sodium Arsenite Intoxication in the Hepato-Renal System of Wistar Rats via the Oxidative Stress Pathway

**DOI:** 10.1155/2022/6178261

**Published:** 2022-12-07

**Authors:** Oluwatoyin O. Ojo, Deborah I. Fatokun, Ikechukwu P. Ejidike, Rachel U. Awolope, Saheed O. Sanni

**Affiliations:** ^1^Department of Chemical Sciences, Faculty of Natural and Applied Sciences, Anchor University, Lagos, Nigeria; ^2^Department of Biotechnology and Chemistry, Faculty of Computer and Applied Sciences, Vaal University of Technology, Vaderbijlpark, South Africa

## Abstract

**Background:**

Chronic exposure to arsenic is a major health concern consequent upon generation of excessive reactive oxygen species. The safety of treatment with chelating agents has not been well established; therefore, there is a need for a paradigm shift in the approach to management of arsenic toxicity. Bioflavonoids are known to influence redox homeostasis in cells; the study therefore investigates the efficacy of quercetin and its zinc and iron metal complexes on sodium arsenite (NaAr)-intoxication in rats.

**Methods:**

Spectroscopic study of quercetin hydrate and its metal complexes was performed using UV-Vis and FT-IR spectrometer. Furthermore, twenty male Wistar rats were obtained and equally divided into four groups, treated orally and daily for 28 days with 10 mg/kg NaAr, 30 mg/kg quercetin, quercetin-zinc, and quercetin-iron separately. Five more rats were used as control. Plasmatic aspartate transferase (AST), alanine transferase (ALT), creatinine (CREA), and total protein (TP) were estimated. Levels of kidney and liver lipid peroxidation (LPO), glutathione (GSH), catalase (CAT), and glutathione-S-transferase (GST) were determined. Histology was used to view the lesions.

**Results:**

Treatment of arsenic-toxicity with quercetin and its complexes decreased the activities of ALT, AST, CREA, TP, CAT, and GST and concentration of LPO and GSH. Quercetin-zn treatment showed a better result than quercetin-iron in the liver. Histology results showed absence of lesions in quercetin zinc and iron treatment in both the kidney and the liver.

**Conclusion:**

Quercetin zinc and iron increased the bioavailability of quercetin and therefore could be relevant as adjuvants in arsenic poisoning.

## 1. Introduction

Environmental pollution remains a major challenge across the globe, especially in developing countries; it is a leading bane in climate change, due to its ability to cause several diseases, thus negatively affecting human health [1]. Exposure to dangerous chemical is among several causes of environmental pollution, these chemicals could be ingested directly or indirectly within the environment and across the food chain [2]. Arsenic is a ubiquitous metalloid harmful in both its organic and inorganic forms, exposure to this chemical will result in multiple organ failure or even death [3–5]. Its toxicity occurs due to consumption of arsenic-contaminated underground water, agricultural outputs, herbicides, and insecticides among others [6]. Toxicity due to arsenic exposure is a global health challenge and more than one million people are at high risk of elevated exposure [3]. Over the years, arsenic intoxication has been managed using chelating agents; however, the therapeutic efficacy has not been established [7, 8]. Consequently, there is a paradigm shift in the approach to the management of arsenic intoxication. Research has shown that oxidative stress-induced reactive oxygen species (ROS) generation plays a crucial role in its pathogenesis [4]. Bioflavonoids have impacted investigation of stress-induced ROS because they have unique characteristic of maintaining a favorable redox balance within the cells. They have also shown the potential to counteract arsenic intoxication induced by free radical generation [9].

Quercetin is a bioflavonoid with numerous health benefits found in many plants and food. It has shown potency in inhibiting arsenic toxicity, however, its metal complexes are assumed to have better activities [10]. Combining quercetin with several metal ions has demonstrated increased reducibility and bioavailability due to conjugates incorporation [10]. We hypothesize that quercetin-zinc and quercetin-iron metal complexes may give a better outcome on the redox status of arsenic-induced toxicity in rat liver and kidney. The use of metal complexes of flavonoids has been employed in pharmaceutical practices; therefore, strategies that would improve the bioavailability and solubility of quercetin are of relevance in medical research. This would enable the optimal exploitation of quercetin as a potent natural antioxidant. The aim of the current work was to investigate the effects of quercetin-zinc (Q-Zn) and quercetin-iron (Q-Fe) on sodium arsenite (NaAr)-induced oxidative stress in the liver and kidney tissues.

## 2. Materials and Methods

### 2.1. Chemicals

Quercetin, NaAr, reduced glutathione (GSH), 5, 5′–dithiobis-2-nitrobenzoic acid (DTNB; Ellman's reagent), and bovine serum albumin (BSA) were purchased from Sigma Aldrich Chemical Co. (St. Louis, MO, USA). Other reagents were of high analytical grade.

### 2.2. Synthesis of Qu-Zn and Qu-Fe Metal Complexes

The complexes were synthesized using a modified procedure as reported by Bukhari et al. [11] Briefly, 4.23 g (0.014 mol) of quercetin hydrate (C_15_H_10_O_7_·xH_2_O) was dissolved in 30 mL of methanol; subsequently, 0.95 g (0.007 mol) of ZnCl_2_ was added rapidly into the reaction mixture and stirred continuously at room temperature for 4 hours. The resulting solution was filtered, and the filtrate was evaporated slowly at 25°C, while the resulting brownish yellow product was washed with t-butanol and dried in a vacuum desiccator. A brownish yellow product, quercetin-Zn (II) complex, was obtained (78% yield). The formation of Qu-Zn complex was confirmed by UV-visible (UV) and Fourier transform infrared (FT-IR).

Similarly, quercetin-Fe (II) complex was synthesized according to Bukhari et al. [11]. Briefly, quercetin hydrate (C_15_H_10_O_7_·xH_2_O) (4.23 g, 0.014 mol) was dissolved in 30 mL methanol within 20 minutes in a 100 mL two-neckedround-bottomed flask equipped with an electromagnetic stirrer and thermometer. Subsequently, FeCl_2_·4H_2_O (1.39 g; 0.007 mol) was added rapidly into the reaction mixture, and the solution was stirred at 25°C for 4 hours. The resulting mixture was filtered and evaporated slowly at room temperature, washed with t-butanol, and dried in a vacuum desiccator. A brownish dark product of 84% yield of quercetin-Fe (II) complex was obtained.

### 2.3. Animals and Treatment

Twenty-five male Wistar albino rats (100–120 g) were obtained from the Animal House of University of Lagos, Idi-Araba, Lagos, Nigeria. They were kept in well ventilated cages under 12 : 12 hour light and dark cycling at 25°C ± 2°C with free access to Purina chow diet and drinking water *ad libitum*.

#### 2.3.1. Body Weight Determination

The body weight of the rats was determined by a weighing balance at the commencement of the study and recorded as the initial weight (g). Subsequently, the weekly weight was determined for each rat and used to adjust the doses of quercetin, Qu-zn, and Qu-iron for standardization throughout the study period.

#### 2.3.2. Treatment

After acclimatization for one week, the rats were randomly divided into 5 groups of 5 animals each. Group I served as the normal control that received fresh water only, while group II, III, IV, and V received drinking water, 10 mg/kg NaAr, 10 mg/kg NaAr + 30 mg/kg quercetin, 10 mg/kg NaAr + 30 mg/kg Qu-Zn, and 10 mg/kg NaAr + 30 mg/kg Qu-Fe metal complex, respectively. All treatments were administered orally, daily, and lasted for 28 days in correlation with previous studies reported by Refat et al. [12]. The animals received care as outlined in the Guide for the Care and Use of Laboratory Animals and Ethics Regulation were strictly adhered to. The dosage of Qu-Zn and quercetin used was based on preliminary investigation of Refat et al. [12]. These were adjusted weekly according to the changes in body weight of the rats.

### 2.4. Sample Collection

Animals were allowed to fast overnight and sacrificed by cervical dislocation. About 3 mL of blood was collected from the beating heart into ethylenediamine tetra-acetic acid tubes. The blood was centrifuged at 3000 ×g for 10 minutes to obtain the plasma. The livers and kidneys were harvested, rinsed, and homogenized in phosphate buffer (0.1 M; pH 7.4). The homogenate obtained was centrifuged at 10000 ×g for 10 minutes (4°C) to obtain the post-mitochondrial fraction (PMF).

### 2.5. Biochemical Assays

The protein concentration of the kidney and liver's PMF were determined by Biuret method according to the procedures of Gornall et al. [13]. Catalase (CAT) activity was estimated at 240 nm according to the method of Claiborne [14]. While Glutathione-S-Transferase (GST) activity was determined according to the method of Habig et al. [15]. 1-chloro-2, 4-dinitrobenzene (CDNB) was used as the substrate and conjugated with GSH and absorption measured at 340 nm. The concentration of GSH was estimated according to the method of Beutler et al. [16]. Briefly, measurement of the yellow product obtained by the addition of DTNB to sulfhydryl compounds was determined at 412 nm. Lipid peroxidation was determined by measurement of the malondialdehyde generated as described by Varshney and Kale [17]. The activities and levels of aspartate aminotransferase (AST), alanine amino transferase (ALT), creatinine, and total protein were determined using AS101, AL 146, CR 2334/MD, and TP 245 Randox kits, respectively (Randox Laboratories Ltd, Crumlin, UK) strictly adhering to the manufacturer's instructions.

### 2.6. Histology Examinations

A part of the kidney and liver tissues were obtained and fixed in 10% formalin solution. The tissues were dehydrated in graded alcohol concentrations and embedded in paraffin. Sections of 5 *μ*m were prepared and stained before microscopic examination and images captured by a digital camera (Leica Biosystems, UK).

### 2.7. Statistical Analysis

Data were analyzed using GraphPad prism 5.0 (San Diego, CA, USA). Multiple comparisons were done by one-way analysis of variance and significance determined by Tukey post hoc test. (Significance was at *P* < 0.05).

## 3. Results

### 3.1. Synthesis and Characterization of Quercetin, Quercetin-Zinc (Qu-Zn), and Quercetin-Iron (Qu-Fe) Metal Complexes

**Figure 1:**
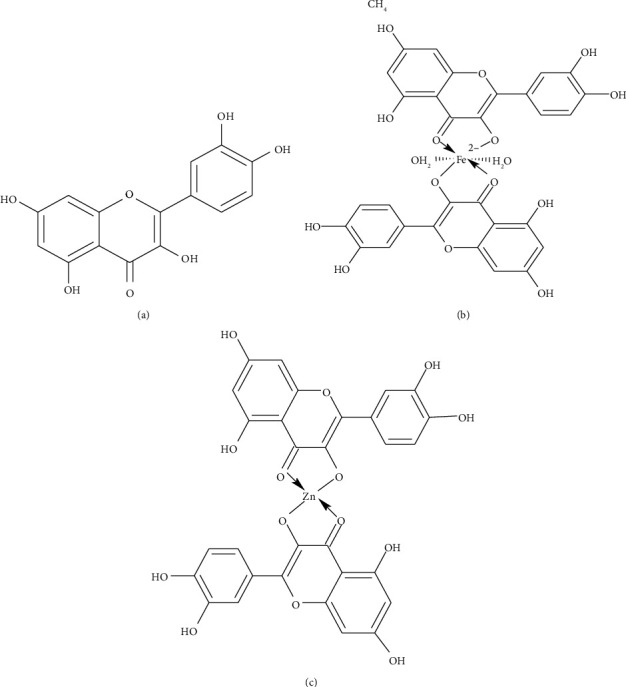
(a) The structure of quercetin; (b) quercetin-zinc (Qu-Zn) metal ion complex; and (c) quercetin-iron (Qu-Fe) metal ion complex.

Figure 1 represents the structure of Figure 1(a) quercetin, Figure 1(b)quercetin-zinc (Qu-Zn) metal complex, and Figure 1(c)quercetin-iron (Qu-Fe) metal ion complex.

**Figure 2:**
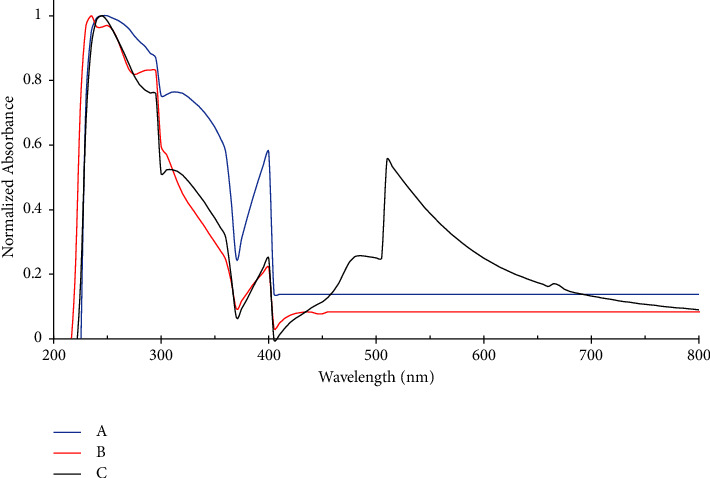
(a) Spectroscopy studies of quercetin (b) quercetin-zinc (c) and quercetin-iron.

The UV-Vis absorption spectroscopy of the complexes is as represented in Figure 2; three characteristics of absorption peaks for quercetin were seen at 250, 315, and 400 nm. These peaks were attributable to *π* − *π*^*∗*^ and *n* − *π*^*∗*^ transition as shown in Figure 2(a). Upon formation of Qu-Zn complex, four absorption peaks were obtained at 235, 250, 295, and 400 nm. The extra peak at 235 indicates the presence of a new bond between the ligand and the metal (Figure 2(b)). Similarly, results for Qu-Fe in Figure 2(c) show the peaks at 250, 295, 310, 400, 485, and 515 nm. The peaks at 295, 485, and 515 nm were due to the formation of Qu-Fe metal complex as shown in Figure 2(c).

**Figure 3:**
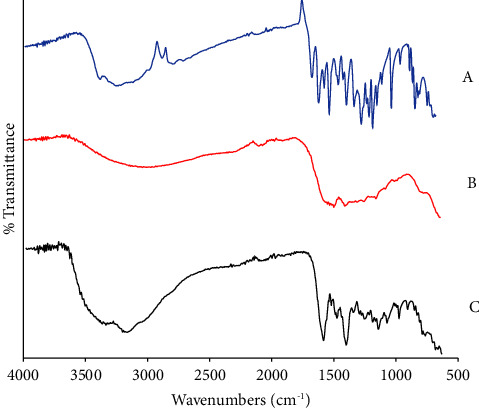
The infrared study of quercetin (a), quercetin-zinc (b), and quercetin-iron (c). The characteristic band of quercetin showing the OH group, CH_2_/CH asymmetry, C=O, and C-O bond was observed in respective complexes.

The functional groups of the complexes were confirmed using FTIR, and results in Figure 3(a) show the infrared study of quercetin. This reveals a characteristic band range from 3385 to 3060 cm^−1^ due to the presence of the OH group. Similarly, another band ranging from 2836 to 2776 cm^−1^ is due to CH_2_/CH asymmetry. Furthermore, the band observed at 1658 cm^−1^ is due to the presence of the carbonyl group (C=O), while those within the range of 1259 and 1010 cm^−1^ is due to the presence of the C-O bond. In Figure 3(b), the OH peak of quercetin became broader due on complexation to zinc, and there was a shift to longer wavelength (3365–2773 cm^−1^). The C=O peak of 1658 cm^−1^ in quercetin disappeared, since it was directly involved in the formation of the complex. There is also a significant change in the C-O bond stretch of the ligand, which showed a broader peak in the Qu-Zn complex. The peak observed at 652 cm^−1^ is due to the zinc-oxygen (Zn-O) bond formed as shown in the Qu-Zn complex. Figure 3(c) shows broad OH peaks observed between 3410 and 3060 cm^−1^, again the broad peak is as a result of the formation of Qu-Fe metal complex, C=O peak was observed at 1602 cm^−1^. The 648 cm^−1^ peak showed the formation of the iron-oxygen (Fe-O) bond.

### 3.2. Effect of Quercetin, Quercetin-Zn, and Quercetin-Fe on the Body Weight Parameters, Kidney, and Liver Functions in Normal and NaAr Intoxication in Rats

The induction of NaAr intoxication in rat led to a sharp drop of about 18.89% in the body weight relative to the normal control (54.63 to 35.74 g) as shown in Table 1. Similarly, treatment of arsenic intoxicated rats with quercetin and Qu-Zn showed a significant difference in the body weight relative to the control rats, while rats treated with Qu-Fe did not significantly differ in the body weight relative to the control rats. Treatment of arsenic intoxication with quercetin, Q-Zn, and Qu-Fe significantly increases the body weights by 8.83, 9.72, and 11.8%, respectively, when compared with arsenic intoxicated rats. There was no significant difference across the treatment groups. Furthermore, assessment of the relative kidney and liver weight showed no differences between NaAr intoxicated rats, control rats, and the treatment groups (Table 1).

### 3.3. Effects of Quercetin, Qu-Zn, and Qu-Fe Metal Complexes on Certain Liver and Kidney Function Tests

**Figure 4:**
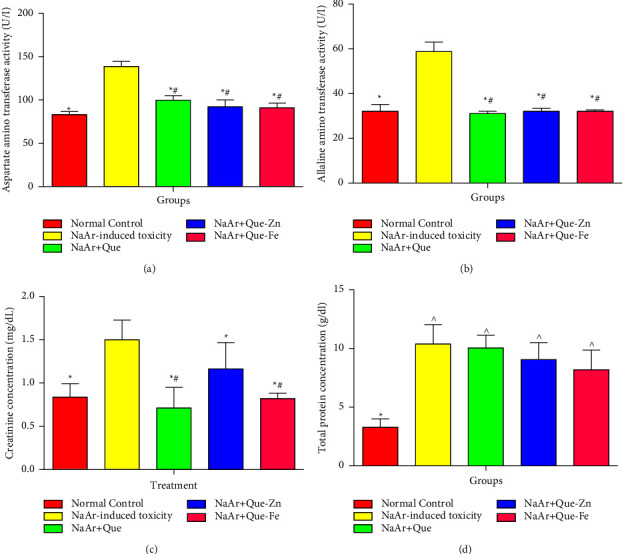
(a) Quercetin and its metal complexes normalized aspartate aminotransferase levels in sodium-arsenite induced toxicity in rats. (b) Quercetin and its metal complexes decreased the elevated alanine amino transferase levels in sodium-arsenite induced toxicity in rats. (c) Quercetin and its metal complexes ameliorate the increased levels of creatinine in sodium-arsenite intoxicated rats. (d) Effect of quercetin and its metal complexes on levels of total protein in sodium arsenite-induced toxicity. Data are presented as the mean ± SD, ^*∗*^ = values differ significantly from untreated sodium arsenite-intoxicated rats (*P* < 0.05), ^#^ = values differ significantly from normal control rats (*P* < 0.05), ^∧^ = values did not differ significantly from untreated sodium arsenite-intoxicated rats (*P* < 0.05). Normal control, NaAr-induced toxicity = rats administered sodium arsenite and left untreated, NaAr + Quer = rats administered sodium arsenite and treated with 30 mg/kg quercetin, NaAr + Quer-Zn = rats administered sodium arsenite and treated with 30 mg/kg quercetin-zinc, and NaAr + Quer-Fe = rats administered sodium arsenite and treated with 30 mg/kg quercetin-iron.

Results in Figures 4(a) and 4(b) show that the plasmatic levels of AST and ALT in NaAr-intoxicated rats were significantly increased (*P* < 0.05) relative to the normal control. Furthermore, treatment of arsenic intoxication with quercetin, Qu-Zn, or Qu-Fe did not show any statistical differences in AST or ALT levels relative to the control rats. Treatment with quercetin, Qu-Zn, and Qu-Fe significantly decreased the levels of AST and ALT in NaAr-intoxication in rats, without any difference within the treatment regimens. Similarly, elevated plasma levels of creatinine and total protein were observed in arsenic intoxication compared with control rats, whereas treatment decreased these levels significantly (Figures 4(c) and 4(d)).

#### 3.3.1. Quercetin and Its Metal Complexes Inhibited Hepatic and Nephrotic Redox State in NaAr-Intoxication in Rats

**Figure 5:**
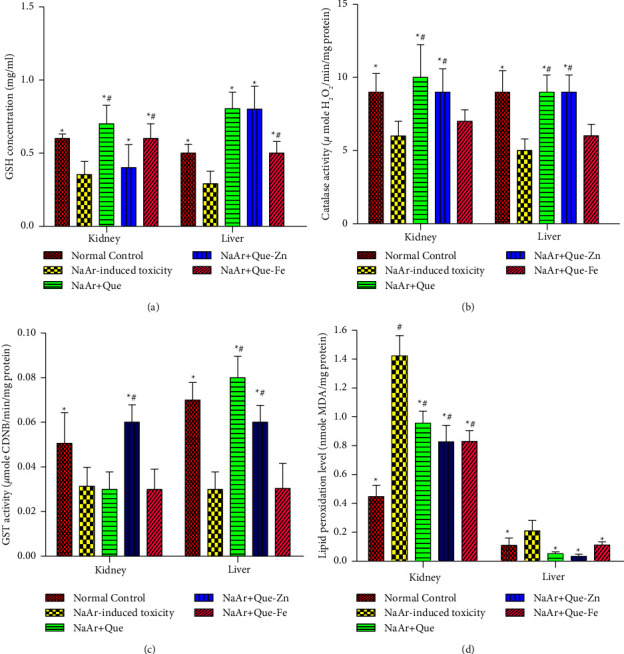
(a) Quercetin and its metal complexes increased glutathione-S-transferase in the liver and kidney of sodium-arsenite induced intoxicated rats. (b) Quercetin and its metal complexes normalized CAT activity in the liver and kidney of sodium-arsenite intoxicated rats. (c) Quercetin and its metal complexes increased glutathione concentration in the liver and kidney of sodium arsenate intoxicated rats. (d) Quercetin and its metal complexes inhibited lipid peroxidation in the liver and kidney of sodium arsenite intoxicated rats. Data are presented as the mean ± SD, ^*∗*^ = values differ significantly from untreated sodium arsenite-intoxicated rats (*P* < 0.05), ^#^ = values differ significantly from normal control rats (*P* < 0.05), normal control, NaAr-induced toxicity = rats administered sodium arsenite and left untreated, NaAr + Quer = rats administered sodium arsenite and treated with 30 mg/kg quercetin, NaAr + Quer-Zn = rats administered sodium arsenite and treated with 30 mg/kg quercetin-zinc, and NaAr + Quer-Fe = rats administered sodium arsenite and treated with 30 mg/kg quercetin-iron.

Figure 5(a) illustrates the effects of Qu-Zn and Qu-Fe on GSH concentrations, CAT and GST in the control, and NaAr intoxicated rat kidney. Arsenic toxicity in rats reduced GSH concentration and CAT and GST activities when compared with control (*P* < 0.05). Furthermore, the GSH concentrations in the treatment of arsenic intoxication with quercetin and Q-Fe were not significantly different from control. Treatment with quercetin and Qu-Fe significantly increased GSH concentration in arsenic intoxicated rats with significant difference only between the groups treated with quercetin and Qu-Zn. The same trend of result was obtained in NaAr-intoxicated rat liver, where there was a significant decrease in the GSH concentration in the arsenic intoxicated rats relative to control. All treatments increased this parameter and comparison within the groups showed a significant difference between quercetin and Qu-Fe (*P* < 0.05). Results in Figure 5(b) show the effect of arsenic intoxication on CAT activity and intervention by quercetin, Qu-Zn, and Qu-Fe. Quercetin and Qu-Zn did not show any differences relative to the normal control, whereas, the activity of CAT was significantly decreased in arsenic intoxication relative to the control rat kidney. Treatment with quercetin and Qu-Zn increased CAT activity with significant difference between quercetin and Qu-Fe treatment. Similarly, in the rat liver, CAT activity was decreased in NaAr intoxication relative to control rats, whereas, all treatments showed an increase in CAT activity more than what was observed in the normal control (*P* < 0.05). Similarly, all treatment regimens increased CAT activity with significant difference between quercetin and Qu-Zn.

The data in Figure 5(c) show that arsenic intoxication presented nephrotic oxidative stress as indicated by decrease in GST activity compared with control. It was also shown that there was no statistical difference between the GST activity in control rats and quercetin treated arsenic intoxicated rats. Whereas, increased activity was observed in treatment with only Qu-Zn (*P* < 0.05), and treatment with quercetin and Qu-Fe did not significantly increase GST activity. Furthermore, it was noted that GST activity in only Qu-Zn treatment significantly differ from quercetin and Qu-Fe treatment (*P* < 0.05). Results in Figure 5(c) show that arsenic intoxication caused decreased GST activity in rat liver relative to control, while treatment with quercetin and Qu-Zn has no significant difference with control. Contrastingly, treatment with all regimens significantly increased the activity with no significant difference within the groups. The result collectively reveals the higher antioxidant potential of quercetin metal complexes relative to quercetin treatment alone.

#### 3.3.2. Effect of Quercetin and Its Metal Complexes on Kidney and Liver Lipid Peroxidation

Results in Figure 5(d) show significant increase in MDA formation in the kidneys and livers of NaAr intoxicated rats relative to control (*P* < 0.05). Treatment of NaAr intoxicated rats with quercetin, Qu-Zn, and Qu-Fe showed a significant difference in the MDA levels relative to control rats. Whereas, these levels were significantly decreased when compared with NaAr intoxicated rats (*P* < 0.05). Similarly, treatments reduced MDA generation in the liver, with the highest efficacy observed in Qu-Zn treatment.

### 3.4. Histopathology

**Figure 6:**
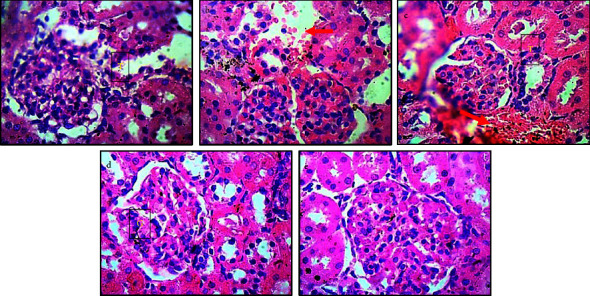
Magnified views of kidney micromorphological section demonstrated by hematoxylin and eosin staining at high magnification (×400). The renal cortex, renal tubules, glomeruli, and mesangial cells as well as proximal and distal renal convoluted tubules are all visible across the various groups. (a) Normal control (normal rats). (b) NaAr-induced toxicity (sodium arsenate-intoxicated rat). (c) NaAr-Quer (sodium arsenate-intoxicated rat + 30 mg/kg quercetin treatment). (d) NaAr-Quer + Zn (sodium arsenate-intoxicatedrat + quercetin-Zn complex treatment). (e) NaAr-Quer + Fe (sodium arsenate-intoxicatedrat + quercetin-Fe complex treatment). Red arrow = degenerative changes characterized with congested proximal and convoluted renal tubules, infiltrated renal parenchyma by red inflammatory cells. *T* = renal tubules, *G* = glomerulus.

Histological examination in control rats shows normal architecture (Figure 6(a)), while Figure 6(b) shows NaAr exposed rats with degenerative changes characterized with congested proximal and convoluted renal tubules, infiltrated renal parenchyma by red inflammatory cells (red arrows). Similarly, the renal tubules were dilated, capsular margin widened, which are signs of glomerulosclerosis, glomerulonephritis, pyknotic renal parenchymal cells, hemorrhage, and concentrated mesangial cells (Figure 6(b)). Treatment with quercetin (Figure 6(c)) reduced the severities in NaAr exposure but did not abrogate it, as degenerative changes were also observed (red arrow). Treatment with Qu-Zn and Qu-Fe showed that the renal cortex had normal glomeruli-, mesangial cells and capsular spaces. The renal tubules appeared normal, clear, and not congested and the interstitial spaces appeared normal with well-defined profile (Figures 6(d)–6(e)).

**Figure 7:**
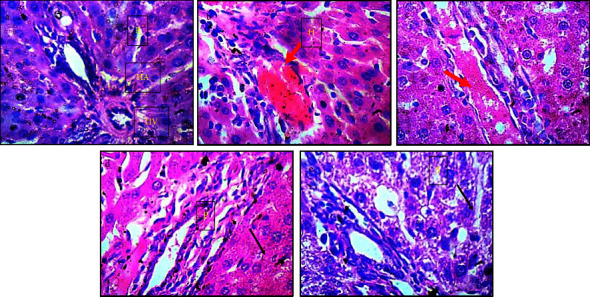
Magnified views of a liver micromorphological section demonstrated by hematoxylin and eosin staining at high magnification (×400). The hepatocytes, sinusoids, and portal triad (hepatic vein, hepatic artery and bile duct) are all visible across the various groups. (a) Normal control (normal rats); (b) NaAr-induced toxicity (sodium arsenate-intoxicated rat); (c) NaAr-Quer (sodium arsenate-intoxicated rat + 30 mg/kg quercetin treatment); (d) NaAr-Quer + Zn (sodium arsenate-intoxicatedrat + quercetin-Zn complex treatment); (e) NaAr-Quer + Fe (sodium arsenate-intoxicatedrat + quercetin-Fe complex treatment). Red arrow = the sinusoids appeared mildly infiltrated and some hemorrhage was observed across the profiles. Black arrow = the sinusoids appearing normal. *B* = bileduct; HA = hepatic artery; HV = hepatic vein; *H* = hepatocytes; *P* = parenchyma; and *S* = sinusoid.

Photomicrographs of the liver sections in control rats showed that the morphology of the cells appeared normal, and the sinusoids were not infiltrated (Figure 7(a)). However, the liver sections of the NaAr-intoxicated rats showed dilated central venules with congestion, the morphology of the hepatocytes present was mildly pyknotic and there was infiltration of the nuclear cytoplasm by fibrosis as well as the presence of inflammatory red cells. The sinusoids appeared mildly infiltrated and some hemorrhage was observed across the profiles as shown by the red arrow (Figure 7(b)). Treatment with quercetin reduced the severity of infiltration by fibrosis as shown in Figure 7(c), but sinusoids were also infiltrated with observed hemorrhage (red arrows). However, Qu-Zn and Qu-Fe treatment restored normalcy in the liver cells of arsenic exposed rats with the sinusoids appearing normal (black arrow) (Figures 7(d) and 7(e)).

## 4. Discussion

Exposure to arsenic is a causative factor for generation of ROS, a prima facie for oxidative stress-induced cell pathogenesis [18]. Until now, chelating agents which causes several health challenges remains the only approach to ameliorating arsenic intoxication. Thus, there is need for a multifactorial approach especially using dietary sources. Quercetin is a polyphenol, present in edible foods including fruits and wine which is nontoxic and biodegradable. Its antioxidant potentials make it relevant in the treatment of oxidative stress associated complications. In this pathophysiology, ROS generation results into altered redox state, which can eventually lead to cell injury or death. The oxidants generated are the key players in oxidative stress-induced tissue damage [19].

In the present study, quercetin was combined with iron and zinc to increase its solubility, bioavailability, and probably induce a synergistic relationship [20]. The new peaks formed on the UV-visible absorption spectra showed that zinc and iron complexes were successfully formed, the respective new functional groups (−OH, −C=O, −–C H), as a result of the new complexes, were also confirmed by FTIR. Quercetin and the metal complexes were used to treat NaAr intoxication in rats for 28 days. Exposure to arsenic caused a loss in body weight, increased activity of hepatic AST and ALT. However, on treating the arsenic intoxicated rat groups with quercetin or its Zn or Fe metal complex, there was weight gain and the activities of AST and ALT were restored to normal values suggesting that maximal antioxidant potential had been attained by all treatments in mitigating the effects caused by liver dysfunction. This observation correlated with the finding of Adil et al. who used naringin to improve the renal and hepatic damage in rats caused by arsenic-induced intoxication [20]. Previous studies have shown that chronic arsenic toxicity could lead to the onset and progression of the liver dysfunction [19]. Similarly, in this study, decreased hepatic GSH content was observed after arsenic intoxication of the induced group, but upon treatment with quercetin, Qu-Zn, and Qu-Fe, normalcy was restored, however, the least effect was observed with Qu-Fe treatment in the liver. Although the CAT and GST activities which are markers for oxidative stress were decreased due to arsenic intoxication in rats, treatment with quercetin, Qu-Zn, and Q-Fe increased the activities. This shows that the complexes neutralize the ROS generation and altered the redox state. The decrease in the activities of CAT and GSH observed in our experiment settings is in line with Yamanaka et al. finding who stated that arsenic initiates ROS production by interaction of arsine specie and ROS to generate a free radical chain of lipid peroxidation [21]. In the present study, nephrotic and hepatic MDA generated in arsenic intoxication may be due to the inability of the cell's inherent antioxidant to cope with the excessive ROS, thereby leading to lipid peroxides generation and eventually cellular damage. Treatment with quercetin and its Zn and Fe metal complexes reduced the MDA generated with Qu-Zn showing the most outstanding results in the liver compared to Qu-Fe. This finding could be due to the fact that the liver is the major site of zinc metabolism [22, 23]. The catalytic, regulatory, and defensive potentials of zinc in cellular metabolism are the probable reasons of the preferential outcomes in the present study. Histology examination showed that the various severities of pathology in the liver sections of arsenic intoxicated rats like dilated central venules, fibrosis, and inflammatory red cells were reduced to mild conditions in quercetin treatment. While Qu-Zn and Qu-Fe showed absence of the observed lesions in arsenic intoxication. This result suggests that the liver has therapeutic preference for Qu-Zn than Qu-Fe, corroborating the suggestions of Bloom et al. [23].

In the study, alteration in the kidney function was observed in NaAr intoxication due to the inability of the organ to clear creatinine, a salient feature of renal function. Treatment with quercetin, Qu-Zn, and Qu-Fe improved glomerular filtration rate via enhanced creatinine clearance. This correlates well with the previous studies of Gholamine et al. who showed that sodium arsenite-induced alteration of hematological and histopathological parameters could be improved upon treatment with gallic acid [5]. Similarly, the increased total protein, an indicator of an underlying injury was observed in NaAr intoxication. Intervention with Qu-Zn and Qu-Fe decreased this effect with the most outstanding result from Qu-Fe, this finding may be linked to the fundamental role renal tissues play an important role in normal iron homeostasis. As this prevents iron loss from the body by reabsorbing it. Exposure of rats to NaAr in the study resulted in decrease renal GSH, CAT, GST, and MDA levels. The decreased GSH concentration in the kidney corroborates well with the study of Das et al. [24]. Similarly, the decreased CAT activity observed in NaAr exposure was increased in the kidney by quercetin and Qu-Zn, alleviating the impediment on the reduction of H_2_O_2_ to H_2_ and O_2_. The decrease in the GST activity observed in NaAr intoxication was normalized by treatment with only Qu-Zn. This would enable GSH remove excess free radicals in the cells, thereby reducing hydroperoxides within membranes and impacting lipid peroxidation. Arsenic intoxication in rats increased the nephrotic MDA generated and all treatment regimens reduced the extent of generation. Histology examinations corroborated the biochemical results with the restoration of normalcy by treatment with Qu-Zn and Qu-Fe in the kidney.

## 5. Conclusion

Given the fact that there is no known treatment for arsenic intoxication except the use of chelating agents, quercetin and its zinc and iron complexes were employed for toxicity management in the present study. Treatment with these regimens decrease oxidative stress in arsenic intoxicated rat kidney and liver and subsequently attenuated severe pathologies in the organs. Quercetin and its zinc and iron metal complexes may therefore have a future role as adjuncts to therapies targeting oxidative stress-induced organ toxicity in arsenic intoxication.

## Figures and Tables

**Table 1:** Effect of quercetin and its complexes on the body weight, relative kidney and liver weight of sodium arsenite-induced alterations in rats.

Groups	% increase in body weight	Relative kidney weight (mg/100 g)	Relative liver weight (mg/100 g)
Normal control	54.63^*∗*^	0.007 ± 0.001	0.037 ± 0.001
NaAr-induced rats	35.74^#^	0.007 ± 0.001	0.037 ± 0.001
NaAr + quercetin	44.07^#^	0.007 ± 0.001	0.035 ± 0.002
NaAr + qquercetin-Zn	45.46^*∗*^^#^	0.007 ± 0.001	0.039 ± 0.004
NaAr + quercetin-Fe	47.54^*∗*^	0.007 ± 0.0015	0.034 ± 0.001

Normal control = normal rats; NaAr-induced rats = sodium arsenate-intoxicated rat; Na-Ar + quercetin = sodiumarsenate-intoxicated rat + 30 mg/kg quercetin treatment; Na-Ar + quercetin-Zn = sodium arsenate-intoxicatedrat + quercetin-Zn complex treatment; Na-Ar + quercetin-Fe. ^*∗*^significant difference with sodium arsenite intoxicated rats, ^#^significant difference with control rats.

## Data Availability

All data used are included in the manuscript and would be made available upon reasonable request.
